# Transcriptome analysis of responses to bluetongue virus infection in *Aedes albopictus* cells

**DOI:** 10.1186/s12866-019-1498-3

**Published:** 2019-06-10

**Authors:** Junzheng Du, Shandian Gao, Zhancheng Tian, Yanni Guo, Di Kang, Shanshan Xing, Guorui Zhang, Guangyuan Liu, Jianxun Luo, Huiyun Chang, Hong Yin

**Affiliations:** 1 State Key Laboratory of Veterinary Etiological Biology, Lanzhou Veterinary Research Institute, Chinese Academy of Agricultural Sciences, Xujiaping 1, Lanzhou, Gansu 730046 People’s Republic of China; 2 Jiangsu Co-innovation Center for Prevention and Control of Important Animal Infectious Diseases and Zoonoses, Yangzhou University, Yangzhou, 225009 People’s Republic of China

**Keywords:** *Aedes albopictus* cells, Bluetongue virus, Transcriptome sequencing, Differentially expressed genes, Vector–virus interaction

## Abstract

**Background:**

Bluetongue virus (BTV) causes a disease among wild and domesticated ruminants which is not contagious, but which is transmitted by biting midges of the *Culicoides* species. BTV can induce an intense cytopathic effect (CPE) in mammalian cells after infection, although *Culicoides*- or mosquito-derived cell cultures cause non-lytic infection with BTV without CPE. However, little is known about the transcriptome changes in *Aedes albopictus* cells infected with BTV.

**Methods:**

Transcriptome sequencing was used to identify the expression pattern of mRNA transcripts in *A. albopictus* cells infected with BTV, given the absence of the *Culicoides* genome sequence. Bioinformatics analyses were performed to examine the biological functions of the differentially expressed genes. Subsequently, quantitative reverse transcription–polymerase chain reaction was utilized to validate the sequencing data.

**Results:**

In total, 51,850,205 raw reads were generated from the BTV infection group and 51,852,293 from the control group. A total of 5769 unigenes were common to both groups; only 779 unigenes existed exclusively in the infection group and 607 in the control group. In total, 380 differentially expressed genes were identified, 362 of which were up-regulated and 18 of which were down-regulated. Bioinformatics analyses revealed that the differentially expressed genes mainly participated in endocytosis, FoxO, MAPK, dorso-ventral axis formation, insulin resistance, Hippo, and JAK-STAT signaling pathways.

**Conclusion:**

This study represents the first attempt to investigate transcriptome-wide dysregulation in *A. albopictus* cells infected with BTV. The understanding of BTV pathogenesis and virus–vector interaction will be improved by global transcriptome profiling.

**Electronic supplementary material:**

The online version of this article (10.1186/s12866-019-1498-3) contains supplementary material, which is available to authorized users.

## Background

Bluetongue (BT) is a major non-contagious disease of ruminants transmitted by biting midges of the *Culicoides* genus. Bluetongue virus (BTV), the etiological agent of BT, is the type species of the *Orbivirus* genus, in the family Reoviridae [1–3]. Historically, the epidemic distribution was limited to tropical and warm temperate regions where the populations of *Culicoides* and the BTV replication cycle were both favored by the warm climate. Since 2006, BTV has spread extensively into several unexpected areas including Southern and Northern Europe, resulting in a serious economic burden [4–7].

A complex non-enveloped virus, BTV has a genome consisting of 10 segments of double-stranded RNA (dsRNA) encoding five different non-structural proteins, NS1, NS2, NS3, NS3A and NS4, as well as seven structural proteins (VP1–7) [8–11]. A BTV particle consists of three successive protein layers which form two capsids. The exterior capsid contains two major structural proteins, VP5 and VP2, while the interior capsid contains another two proteins, VP3 and VP7, and encloses a viral transcription complex composed of VP1 (polymerase), VP4 (capping enzyme), and VP6 (helicase) proteins, as well as the viral genome [8, 10, 12, 13]. The non-structural proteins are mainly involved in virus assembly, replication, trafficking, release and morphogenesis [9–11, 14].

The transcriptome is a whole set of gene transcripts of specific cells, tissues, organs, or complete organisms, which associates the genetic information of the genome and the biological function of the proteome. The interaction between hosts or mammalian cells and pathogens such as Marek’s disease virus, influenza virus, avian leukosis virus subgroups, bovine viral diarrhea virus, avian infectious bronchitis virus, Schmallenberg virus and tick-borne flaviviruses has been studied previously by transcriptome analysis [15–21]. Recently, deep sequencing has been considered to be a potent approach to transcriptome analyses which is superior to conventional methods in terms of repeatability and the false-positive rate, as well as the dynamic scale [22, 23]. In this study, we used *Aedes albopictus* cells to reveal the transcriptome changes after infection with BTV, given the lack of the *Culicoides* genome sequence. Following this, several mRNA transcripts were selected to confirm the sequencing data by quantitative reverse transcription–polymerase chain reaction (qRT-PCR). The global transcriptome profiling will provide a deep understanding of BTV pathogenesis and virus–vector interactions.

## Methods

### Cells and virus

*A. albopictus* cells (ATCC-CCL-126) and BHK-21 cells (ATCC-CCL-10) were used in this study. *A. albopictus* cells were cultured in Dulbecco’s modified Eagle’s medium (DMEM, HyClone, USA) with the addition of 10% fetal bovine serum (FBS) (Gibco, USA) at a temperature of 28 °C. BHK-21 cells were cultured in modified Eagle’s medium (MEM, HyClone) with the addition of 10% FBS at 37 °C with an atmosphere containing 5% CO_2_. A BTV-1 strain (GS/11), which was isolated from sheep in western China in 1997, was propagated in BHK-21 cells and was used for viral infection. A BHK-21 monolayer was utilized to determine the virus titer, using the plaque formation assay [13].

### Immunofluorescence

*A. albopictus* cells were seeded in 12-well plates and grown on glass coverslips (NEXT, China) and subsequently infected with BTV (multiplicity of infection (MOI =1)) and incubated for 12 or 24 h. Cells were then fixed with 4% paraformaldehyde (Solarbio, China), and permeabilized with 0.05% Triton X-100. BTV-infected fixed cells were incubated with a rabbit polyclonal antibody (1:1000) against recombinant BTV NS1 protein expressed in *Escherichia coli* (*E.coli)* and, subsequently, with the Alexa Fluor 568 anti-rabbit secondary antibody (Abcam, UK) (1:3000). Nuclei were counterstained with Hoechst 33258 (Invitrogen, USA). Cover glasses were mounted on glass slides using fluorescence mounting medium (ZSGB, China). Images were obtained using a fluorescence microscope (Leica, Germany).

### Western blot

BTV-1 infection in *A. albopictus* cells was also confirmed by western blot analysis. BTV-infected and mock-infected cells in 12-well plates were harvested at 12 and 24 h post infection (hpi) with a cell scraper, separated at 1000×*g* in a centrifuge (Eppendorf 5424 R, Germany) for 5 min, and the cell lysate pellet washed three times with phosphate-buffered saline (PBS). Cell lysates were denatured in 1 × protein loading buffer (10 mM Tris-HCl, pH 8.5, 50 mM DTT, 1% SDS, 10% glycerol, and 0.008% bromophenol blue) by heating for 5 min at 100 °C. SDS-PAGE was utilized to separate the proteins in the cell lysates, which were subsequently transferred onto nitrocellulose membranes (Millipore, USA). A blocking solution (0.5% Tween-20 and 5% skimmed milk) was utilized to block the membranes for 1 h. Rabbit polyclonal antibodies against recombinant BTV-1 NS1, NS2, and VP6 proteins expressed in *E.coli* in our laboratory, were used for probing, after which membranes were incubated with goat anti-rabbit IgG H&L (alkaline phosphatase) secondary antibody (Abcam, UK).

### Virus infection

To avoid contamination of BHK-21 cell debris, the virus used in this study was passaged three times in *A. albopictus* cells and then centrifuged after three freeze-thaws to remove the cell lysates. The supernatant was used to measure virus titers and to infect *A. albopictus* cells. *A. albopictus* cells were infected with BTV as described previously [24]. Briefly, to characterize the transcriptome profiles of *A. albopictus* cells after infection with BTV, 3 × 10^6^ cells in 25-cm^2^ flasks (Corning, USA) were infected in three replicates with a MOI of 1. The cells were adsorbed with virus for 1 h at room temperature and then cultured in DMEM with the addition of 2% FBS. *A. albopictus* cells without virus infection were used as the mock-infected group, in three replicates.

### RNA extraction and transcriptome sequencing

BTV-infected and -mock-infected cells were collected at 24 hpi with a cell scraper, separated at 1000×*g* in a centrifuge (Eppendorf 5810 R, Germany) for 5 min, and the pellet washed three times in ice-cold PBS. Total RNA was extracted using a mixture of three replicated samples of cells using TRIzol reagent (Invitrogen, USA) and then digested with DNase I enzyme (TaKaRa, Japan). Oligo (dT) magnetic beads were utilized for poly (A) mRNA isolation, which was subsequently digested into fragments as a template for synthesizing first- strand cDNA using random primers and reverse transcriptase. RNase H, dNTPs, and DNA polymerase I were used to synthesize second-strand cDNA, which was then purified, repaired at the ends, connected with sequencing adaptors, and amplified by PCR to create a cDNA library using the Truseq™ RNA Sample Prep Kit (Illumina, USA). The library was evaluated with an Agilent 2100 Bioanalyzer (Agilent Technologies, USA) and StepOne Plus Real-time PCR System (Applied Biosystems, USA), and was sequenced using an Illumina HiSeq™ 2000 sequencer (Illumina). The aforementioned RNA samples were utilized for qRT-PCR analysis of selected mRNA transcripts.

### Deep sequencing analyses

Firstly, empty reads and adaptors, as well as reads filtered for low quality, were removed. Secondly, the reads were mapped to the *A. aegypti* genome using Bowtie software [25]. Lombardo et al. reported that several of the transcripts identified in *A. albopictus* showed a good level (70–100%) of similarity with their *A. aegypti* homologs [26]. RNASeq by Expectation Maximization (RSEM) software (http://deweylab.biostat.wisc.edu/rsem/) was utilized to analyze differentially expressed genes and to quantify transcripts [27]. The filtering standard for the data was a false-discovery rate-corrected *P* value (*q* value) < 0.001 and a fold- change > 2. The databases local BLAST, Cluster of Orthologous Groups (COG), STRING and SwissProt were applied to predict and annotate all unigenes. The unigenes were analyzed by the Blast2GO tool on the basis of Gene Ontology (GO) terms. The mRNAs exhibiting differential expression were entered into the databases UniProt and the integrated discovery (DAVID) online server (http://david.abcc.ncifcrf.gov) to be annotated and visualized. The analyses included classifications of cell constituents and molecular function, as well as biological processes, with a confidence level of 95%. The mRNA transcripts identified were grouped and classified by Kyoto Encyclopedia of Genes and Genomes (KEGG) pathway analysis. The STRING 10 database (http://string.embl.de/) was utilized to analyze the network of interactions between proteins, based on the identified mRNA transcripts [28].

### Validation of sequencing data

qRT-PCR was used for the detection of several selected mRNAs with differential expression, with the aim of confirming the data of RNA-Seq. The afore-described protocol was used to prepare total RNA, which was subsequently digested using DNaseI enzyme (Promega, USA). The Mx3500p system (Agilent Technologies) was applied to perform qRT-PCR. First-strand cDNAs were synthesized using the PrimerScript RT Master Mix (TaKaRa, Japan). A SYBR Premix Ex Taq™ kit (TaKaRa) was used to perform real-time PCR, following the manufacturer’s instructions. The PCR program was set as: 95 °C for 30 s, then 38 cycles of 95 °C for 5 s and 60 °C for 20 s. Table 1 shows the respective primer sequences of the reference gene β-actin (*A. albopictus*) and selected mRNA transcripts. Each assay was conducted in three replicates. The method of 2^—ΔΔCT^ was used to calculate the relative expression levels of mRNAs in cells after BTV infection, which were expressed as the relative fold-change in the expression level in infected cells divided by that in control cells [29].

**Table 1:** Primer sequences for analysis of gene expression using qRT-PCR

Primer name	Sequence (5′-3′)	Product size (bp)
AAEL015390-F	AGGCGAAAGCCAAAGCAGTT	90
AAEL015390-R	TCGTGCGGTTCTTCAGGTGT	
AAEL010126 -F	TCCACCTCGTCGTCACCTTG	131
AAEL010126-R	TGTTCGTCGTAGCTGTCGCT	
AAEL009532-F	CTGCTGTTCCACACGCTGAC	189
AAEL009532-R	TAACCGCGCTCTCCGAATGT	
AAEL001603-F	GCTGCCCATCCAGAACAAGC	163
AAEL001603-R	GGTGCGGCCACTGTATGTTG	
AAEL012071-F	GCGCGTCAAAGATGCAGAGG	166
AAEL012071-R	GAAGGCATCGTCGACTCCCA	
AAEL001165-F	ACAGTTCGGCCAACTCGTCA	149
AAEL001165-R	ACTGGCTGTTGGTGACTGCT	
AAEL002903-F	TTTGCGCACCATCCAAGACG	139
AAEL002903-R	GTGCCCAATGTGCTGGTTGT	
AAEL004715-F	CGACGGGTAGCAGTAGCTGT	150
AAEL004715-R	TGTTGTGCTTGCTCTGCGTT	
AAEL010488-F	CCGTCTCCCAGTCACCTGTC	163
AAEL010488-R	CGTCATCCTGTTGGCTGTGC	
β - actin-F	GGAGAAGATCTGGCATCACA	95
β - actin-R	TGTCATCTTCTCGCGGTTAG	

## Results

### BTV infection in *A. albopictus* cells

**Fig. 1:**
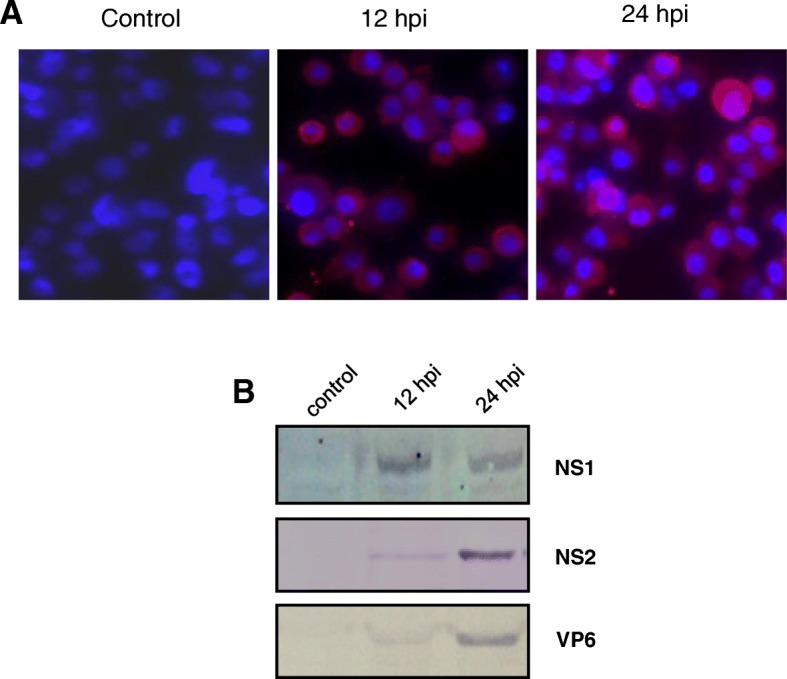
Confirmation of BTV replication in *Aedes albopictus* cells. **a** Immunofluorescence assay (NS1 protein) of BTV-infected cells at 12 and 24 hpi. **b** Western blot analysis of NS1, NS2, and VP6 in BTV-infected and control samples at 12 and 24 hpi

To examine the transcriptomic responses, BTV-1 was used to infect *A. albopictus* cells with a MOI of 1 to ensure a high ratio of virion to cells. As described previously, no morphological changes occurred in *A. albopictus* cells infected with BTV, which led to non-lytic infection without cytopathic effect (CPE) [24]. To confirm viral replication in *A. albopictus* cells following BTV infection, immunofluorescence, and western blot assays were performed using antibodies against NS1, NS2, and VP6 proteins. The immunofluorescence assay showed that NS1 was found in the cytoplasm and almost all cells were positive for BTV NS1 expression at 12 and 24 hpi (Fig. 1a). The NS1, NS2, and VP6 proteins were also detected at 12 and 24 hpi by western blot (Fig. 1b). These results confirmed BTV replication in *A. albopictus* cells, although no CPE appeared.

### Transcriptome alteration in *A. albopictus* cells infected with BTV

**Fig. 2:**
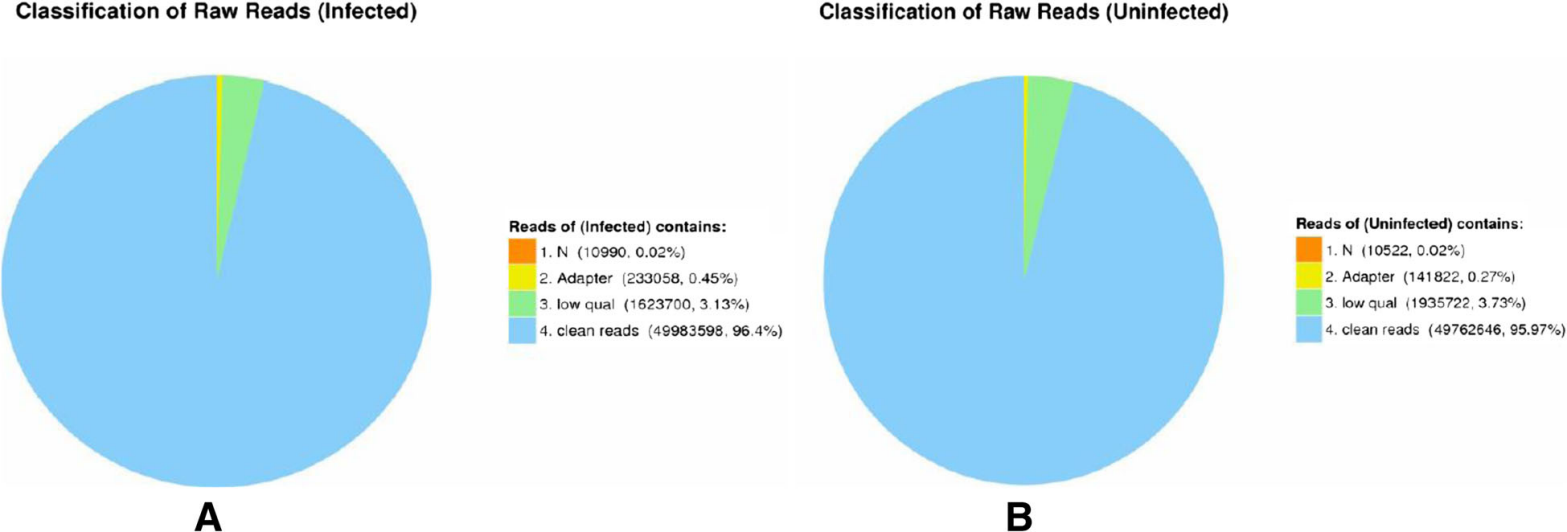
Classification of raw reads from BTV-infected (**a**) and uninfected (**b**) *Aedes albopictus* cells

**Fig. 3:**
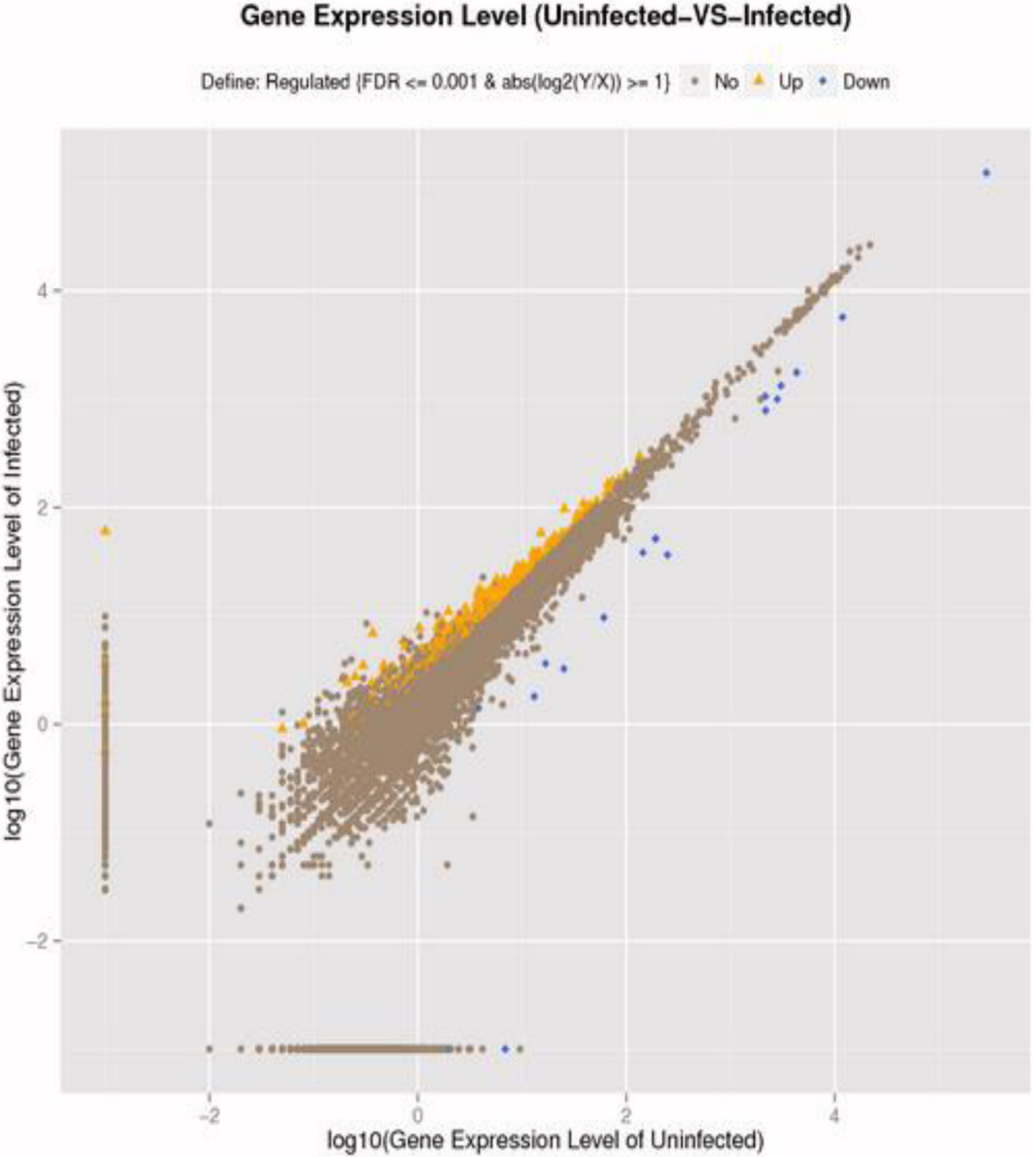
Statistical charts of all expressed genes in BTV-infected and uninfected groups. The *x* and *y* axes represent the levels of expression of the mRNAs of the two groups. The yellow triangles represent mRNAs up-regulated in BTV-infected cells, and the blue points represent mRNAs down-regulated in BTV-infected cells

To identify host cell transcripts involved in BTV-1 infection, libraries of mRNAs from BTV-infected and -mock-infected *A. albopictus* cells at 24 hpi were submitted to high-throughput sequencing. In total, 51,850,205 raw reads were generated from the BTV infection group and 51,852,293 from the control group. After empty reads and adapter sequences, as well as sequences of poor quality, were removed, 49,983,598 clean reads were obtained from the infection group and 49,762,646 from the control group (Fig. 2). In total, 12,822,376 clean reads in the infection group and 14,240,610 in the control group were completely mapped to the *A. aegypti* genome by Bowtie software. The majority of unigenes (5769; accounting for 80.6% of the total unigenes) were represented in both infection and control groups. However, 779 unigenes, which accounted for 10.9% of the total unigenes, existed exclusively in the infection group, while 607 unigenes, which accounted for 8.5% of the total unigenes, existed exclusively in the control group (Additional file 1). Differential expression analyses were performed using RSEM, and genes with a fold-change value ≥2 (∣log_2_ fold change∣ ≥ 1) and *q* values < 0.001 were accepted as significant. A total of 380 differentially expressed genes were detected, which indicated that the genes were related to BTV infection (Fig. 3, Additional file 2). Among the mRNA transcripts with differential expression, the expression of 362 genes was up-regulated, with fold differences ranging from 15.9- to 1-fold threshold value, and 18 genes were down-regulated, with fold differences ranging from − 12.8- to − 1-fold threshold value (Additional file 2).

### Bioinformatics analyses

**Fig. 4:**
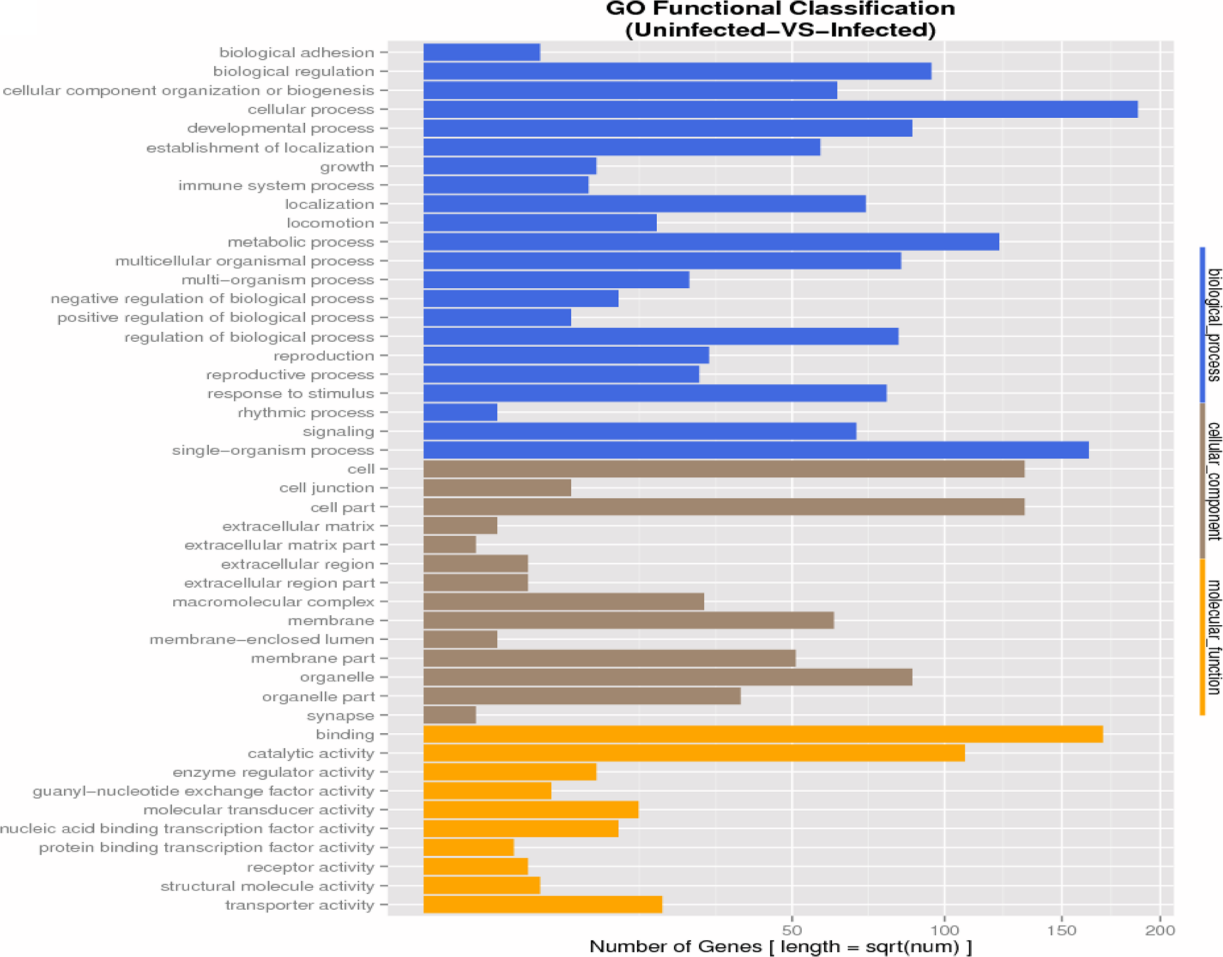
GO pathway enrichment analysis of 380 differentially expressed genes based on their functional annotations, including 603 terms for biological processes, 98 for cellular components, and 129 for molecular functions

To further examine the biological functions of the 380 differentially expressed genes in *A. albopictus* cells upon BTV infection, GO terms were used to classify the functions of the BTV-infected cell transcripts, producing 603 terms for biological processes, 98 for cellular components, and 129 for molecular functions (Additional file 3). The annotation of biological processes indicated that most of the proteins under differential regulation were related to cellular and developmental processes, localization, biological regulation, metabolic processes and response to stimulus. It was shown by the annotation of cell constituents that most of the proteins with differential expression profiles were uniformly distributed in a variety of cellular constituents, including membranes, organelles and macromolecular complexes. It was revealed by the molecular function annotation that virus-infected cells most frequently had changes in functions relating to binding and catalytic activity, as well as transporter function (Fig. 4). The KEGG pathway analyses showed that the genes with differential expression participated principally in endocytosis, FoxO, MAPK, dorso-ventral axis formation, insulin resistance, JAK-STAT, and Hippo signaling pathways (Additional file 4).

**Fig. 5:**
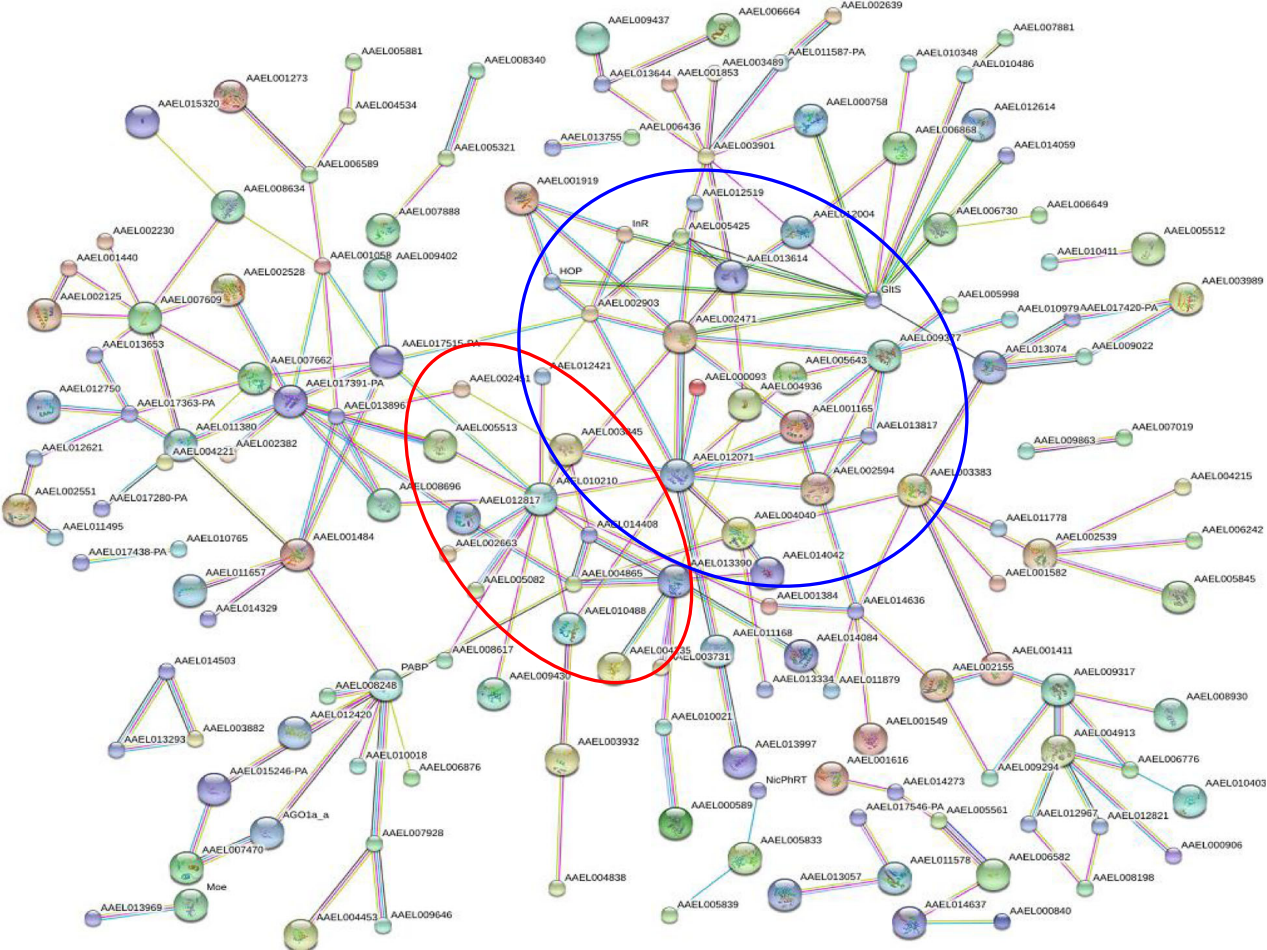
Interaction network of differentially expressed genes generated using the STRING database. The edges represent predicted functional associations. An edge was drawn with up to seven differently colored lines representing the existence of seven types of evidence used in predicting the associations. The red lines indicate fusion evidence, the green lines indicate neighborhood evidence, the blue lines indicate co-occurrence evidence, the purple lines indicate experimental evidence, the yellow lines indicate textmining evidence, the light-blue lines indicate database evidence, and the black lines indicate co-expression evidence

To clarify protein–protein interaction networks, the STRING database was searched to analyze further the 380 genes with differential expression. As shown in Fig. 5, several groups of proteins with strong interactions under significant regulation by BTV were detected, including AEL012071-AAEL002471-AAEL003845-AAEL000093-AAEL002594-AAEL004040 and AAEL010210-AAEL012421-AAEL010488-AAEL002663-AAEL005082-AAEL005513. The above proteins have an essential role in innate immunity. The influence of BTV on the physiological function of infected cells was further clarified by analysis of interactive connections under potential regulation by BTV.

### Validation of differentially expressed mRNAs

**Fig. 6:**
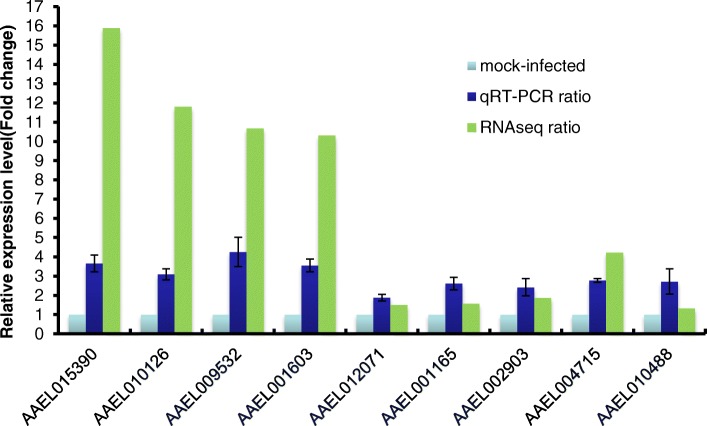
qRT-PCR validation of nine selected mRNAs. The relative expression level of each mRNA transcript in BTV-infected cells was calculated using the 2^—ΔΔCT^ method and represented as the *n*-fold change relative to the uninfected cells

Transcriptome sequencing yields a large amount of data, and it is important to validate differential expression by independent methods. In order to confirm the results of the transcriptome sequencing, genes with differential expression were detected using qRT-PCR. Nine genes (AAEL015390, AAEL010126, AAEL009532, AAEL001603, AAEL012071, AAEL001165, AAEL002903, AAEL004715 and AAEL010488), which exhibited significant alterations in expression profiles after BTV infection, were validated using qRT-PCR. The result revealed that the relative expression levels of mRNAs of AAEL015390, AAEL010126, AAEL009532, AAEL001603, AAEL012071, AAEL001165, AAEL002903, AAEL004715 and AAEL010488 increased 3.66-, 3.09-, 4.26-, 3.56-, 1.89-, 2.61-, 2.42-, 2.78-, and 2.72-fold, respectively, in BTV-infected *A. albopictus* cells compared with the mock-infected cells at 24 hpi (Fig. 6), which conformed to the results of transcriptome sequencing, although there were fold differences between the qRT-PCR and transcriptome data, indicating that the expression profiles of differentially expressed genes in *A. albopictus* cells were notably changed in response to BTV infection.

## Discussion

The infection of final hosts caused by arboviruses usually exhibits acute and pathogenic properties, while that in vectors is relatively moderate and non-pathogenic [3, 30]. It is well known that biting midges of the *Culicoides* genus are able to carry and transmit arboviruses, some of which result in infection among animals throughout the world, for example African horse sickness virus and BTV, as well as Schmallenberg virus, which was discovered recently [3, 7, 31, 32]. BTV can induce a strong CPE in mammalian cells after infection while *Culicoides*- or mosquito-derived cell cultures cause non-lytic infection without obvious CPE [24, 33–36]. The study of interactions between arboviruses and *Culicoides* vectors has been restricted by the absence of *Culicoides* genome sequences [37, 38]. *A. albopictus* cells are derived from mosquitos and are usually used for studies of BTV and other arboviruses [39–42]. Nevertheless, data on alteration of the transcriptome of *A. albopictus* cells in response to BTV infection were previously unavailable. In this study, the transcriptome was sequenced for the identification of the mRNA expression pattern in BTV-infected *A. albopictus* cells. In total, 12,822,376 and 14,240,610 clean reads were obtained from cells with and without BTV infection, respectively. A total of 380 differentially expressed genes (362 up-regulated and 18 down-regulated) were identified in the study, which strongly indicated that the differentially expressed genes are involved in BTV infection. Production of mature viral particles was exponential at 8 and 24 hpi [1]. With antiviral response in mind, we focused on transcriptome changes during the early stage of infection (24 hpi), which avoided RNA degradation and interference with cell maintenance at the late stage of infection in order to guarantee the quality of cDNA libraries for transcriptome sequencing. While our manuscript was in preparation, the genome of *Culicoides sonorensis,* a vector of BTV, was sequenced, which will facilitate the identification of potential antiviral factors and unravel the transmission mechanism of BTV as well as other arboviruses [43]. Soon, we will investigate the alterations in the transcriptome of *Culicoides sonorensis* (KC) cells infected with BTV to see how the changes induced by BTV infection of KC cells differ from those in *A. albopictus* cells.

In insects, the infectious outcomes are notably influenced by the interactions between viruses and the innate immunity of the vectors, in spite of the evidence that the immune response of insects is similar to adaptive immunity in mammals [38, 44]. One of the principal mechanisms of defense against viruses is RNA interference (RNAi), which inhibits viral replication by detecting dsRNA derived from viruses [44, 45]. We recently performed deep sequencing to identify micro RNAs (miRNAs) with differential expression in BTV-infected *A. albopictus* cells. The results showed that 140 miRNAs with differential expression, including 125 novel candidates and 15 known miRNAs, were detected and predicted to be essential regulatory miRNAs in the early stage of BTV infection [24]. In addition to RNAi, other innate antiviral pathways such as Toll and JAK-STAT were also revealed as essential regulators of insect antiviral responses [44, 46–48]. Viruses, fungi, and Gram-positive bacteria are principal activators of the Toll pathway, which, to a great extent, controls antimicrobial peptide (AMP) expression [49]. The JAK-STAT pathway was originally identified in mammals, and proved to play an essential role during infection by viruses such as dengue virus and *Drosophila* C virus [50, 51]. Moreover, this pathway was found to be conserved in defense against viruses among insects and human beings [52, 53]. In this study, the JAK-STAT signaling pathway was also identified among the differentially expressed genes, but the Toll signaling pathway was not identified, indicating that the JAK-STAT and Toll pathways act in two distinct antiviral networks. This result is consistent with a recent report from our laboratory on miRNA expression analysis in BTV-infected *A. albopictus* cells [24]. These findings strongly indicate that the JAK-STAT pathway may have an important action in BTV–vector interaction.

Endocytosis provides pathways through which many viruses productively infect their target cells [54]. Different mechanisms are available for the endocytic internalization of BTV particles, including clathrin-mediated endocytosis and macropinocytosis [13, 55–57]. Herein, the results showed that several of the differentially expressed genes may be involved in endocytosis, which provides a possible insight into the pathway of BTV entry into *A. albopictus* cells. From our current data, it is not clear so far how this possible role of the differentially expressed genes would favor the overall infection of the *A. albopictus* cells by endocytic pathway. One possibility inviting speculation in this point is that the fraction of cells that are actually infected by BTV (note that our use of MOI of 1, does not warrant infection of the totality of the cells) would somehow be up-regulating the endocytic pathways in the yet-to-be-infected cells. BTV is capable of infecting a variety of cells, including HeLa, BHK-21, MDBK, KC and *A. albopictus* cells, and probably infects host mammalian cells and vectors through various mechanisms [13, 34, 55, 57]. The exact mechanism by which BTV infects *A. albopictus* cells requires further investigation.

Programmed cell death (PCD), also known as apoptosis, acts as an intrinsic response to viral infection, which is able to limit viral replication and growth in mammalian cells [58, 59]. The replication of some arboviruses were also shown to be suppressed in a PCD manner in insect cells after infection [60–62]. Our results would suggest that the Hippo and FoxO signaling pathways, which are known to participate in PCD, were identified as differentially expressed in *A. albopictus* cells, which is consistent with our previous report [24]. These results are really intriguing at this time and future efforts need to be directed towards ascertaining whether Hippo and FoxO signaling pathways actively inhibit BTV replication in *A. albopictus* cells.

## Conclusion

In summary, we investigated the alteration in the expression of mRNA transcripts with differential expression in BTV-infected *A. albopictus* cells by transcriptome sequencing. A total of 380 differentially expressed genes were detected, 362 of which were up-regulated and 18 of which were down-regulated. Bioinformatics analyses showed that the differentially expressed mRNAs were mainly involved in endocytosis, FoxO, MAPK, dorso-ventral axis formation, insulin resistance, and the Hippo and JAK-STAT signaling pathways. Consequently, these differentially expressed mRNAs probably play an essential role in antiviral immune responses and viral pathogenesis in insects and insect cells. The results of this study may be helpful in identifying potential antiviral factors and providing molecular clues for unraveling the mechanism of non-lytic BTV infection, and that involving other arboviruses.

## Additional files

Additional file 1: List of unigenes identified in BTV-infected and uninfected cells. (XLSX 1431 kb)

Additional file 2: List of differentially expressed genes. (XLSX 99 kb)

Additional file 3: GO annotations for differentially expressed genes. (XLS 394 kb)

Additional file 4: KEGG Pathway annotations for differentially expressed genes. (XLS 25 kb)

## Data Availability

All sequencing data generated in this study have been deposited at the Sequence Read Archive (SRA) database at National Center for Biotechnology Information (NCBI) under the accession number PRJNA436549.

## Abbreviations

AMP: Antimicrobial peptides
BT: Bluetongue
BTV: Bluetongue virus
CPE: Cytopathic effect
DAVID: Databases UniProt and the integrated discovery
DMEM: Dulbecco’s modified Eagle’s medium
dsRNA: Double-stranded RNA
FBS: Fetal bovine serum
GO: Gene Ontology
hpi: Hours post infection
KC: *Culicoides sonorensis*
KEGG: Kyoto Encyclopedia of Genes and Genomes
MEM: Modified Eagle’s medium
MOI: Multiplicity of infection
PBS: Phosphate-buffered saline
PCD: Programmed cell death
PCR: Polymerase chain reaction
PFU: Plaque formation units
qRT-PCR: Quantitative reverse transcription–polymerase chain reaction
RSEM: RNASeq by expectation maximization
